# Icariin promotes the repair of PC12 cells by inhibiting endoplasmic reticulum stress

**DOI:** 10.1186/s12906-021-03233-1

**Published:** 2021-02-19

**Authors:** Chengjie Wu, Guanglu Yang, Yalan Pan, Lei Wang, Pengcheng Tu, Suyang Zheng, Yang Guo, Yong Ma

**Affiliations:** 1 Department of Traumatology and Orthopedics, Affiliated Hospital of Nanjing University of Chinese Medicine, Nanjing, 210029 People’s Republic of China; 2 Laboratory of New Techniques of Restoration & Reconstruction, Institute of Traumatology & Orthopedics, Nanjing University of Chinese Medicine, Nanjing, China; 3 Laboratory of Chinese Medicine Nursing Intervention for Chronic Diseases, Nanjing University of Chinese Medicine, Nanjing, China

**Keywords:** Endoplasmic reticulum stress, Neuron, Icariin, CHOP, Grp78

## Abstract

**Background:**

Endoplasmic reticulum stress (ERS) is one of the main mechanisms of spinal cord injury (SCI) pathology and can affect the physiological state of neurons. Icariin (ICA), the main pharmacological component of Epimedium, can relieve the symptoms of patients with SCI and has obvious protective effects on neurons through ERS.

**Methods:**

PC12 cells were induced to differentiate into neurons by nerve growth factor and identified by flow cytometry. Cell proliferation was detected by CCK8 method, cell viability was detected by SRB assay, apoptosis was detected by flow cytometry and microstructure of ER was observed by transmission electron microscope. Western blot was used to detect the protein expression of CHOP and Grp78, and qPCR was used to detect the mRNA expression of CHOP and Grp78.

**Results:**

The results of CCK8, SRB and flow cytometry showed that ICA could relieve ERS and reduce apoptosis of PC12 cells. The results of transmission microscope showed that ICA could reduce apoptosis of PC12 cells caused by ERS. The results of Western blot and q-PCR showed that ICA could inhibit ERS by down-regulating the expression of CHOP and Grp78.

**Conclusions:**

ICA can inhibit ERS and promote the repair of PC12 cells by down-regulating the expression of CHOP and Grp78. ICA has the potential to promote the recovery of spinal cord injury.

**Supplementary Information:**

The online version contains supplementary material available at 10.1186/s12906-021-03233-1.

## Background

Spinal cord injury (SCI) is one of the most severely damaged diseases of the central nervous system, which can lead to loss of sensory, motor function and a decline in quality of life [1]. As the population base increased, the total number of cases of SCI also increased significantly. Among them, the incidence of elderly patients increased the most, and the hospital mortality rate was still high [2]. Although recent studies have partially elucidated the pathophysiological processes after SCI, in addition to conventional SCI treatment and rehabilitation, innovative and effective treatment options have been still limited [1].

Modern pharmacological research and clinical practice have proved that Herba Epimedii and its active compound (icariin) have a wide range of pharmacological effects, especially in hormone regulation, anti-osteoporosis, immune function regulation, anti-oxidation, anti-aging [3]. Icariin (ICA) could attenuate AGE-induced oxidative stress and mitochondrial apoptosis by specifically targeting Bax and further regulating the biological function of Bax on mitochondria [4]. Some studies have shown that Icariin protected endoplasmic reticulum stress (ERS)-induced apoptosis of PC12 cells in a Synoviolin-dependent manner [5].

When cells are exposed to external stress factors and structural abnormalities, unfolded proteins accumulate in ER, triggering the ERS pathway. It can activate the pro-apoptotic factor, C/EBP homologous transcription factor protein (CHOP), which mediates programmed cell death. Glucose-regulated protein 78 (GRP78) is the molecular chaperone of ER. In order to protect cells from unfolded proteins, it can mediate the refolding of unfolded proteins [6]. ER has the function of regulating transmembrane protein and intracellular calcium concentration, synthesizing phospholipids and cholesterol, and affecting protein folding [7]. However, ERS reaction occurs in the body under the conditions of hypoxia, ischemia and trauma, which is characterized by protein folding and unfolded protein accumulation in the ER lumen [8]. ERS is one of the main mechanisms of SCI pathology [9], which can induce cell apoptosis by activating CHOP [10]. However, the three ERS sensors, inositol-requiring enzyme 1 (IRE-1), activating transcription factors 6 (ATF6) and protein kinase-like ER kinase (PERK) in the cell can alleviate ERS [11, 12], eliminate misfolded or unfolded proteins, and return the cell to steady state [13]. In normal cells, the chaperone protein Grp78 binds to the ER membrane protein PERK and blocks its activation. When unfolded proteins accumulate under ERS, Grp78 dissociates from PERK, leading to its activation, which inhibits protein synthesis and ultimately reduces the overload of misfolded proteins in ER [10, 14]. Due to the existence of internal ribosome entry sites in its mRNA, the translation of some mRNA (such as ATF4) is not inhibited, and ATF4 protein can increase the expression of genes involved in protein folding and redox control [15, 16]. If ERS reacts violently, ATF4 will increase the expression of CHOP and induce apoptosis [17].

Therefore, CHOP and GRP78 are the key factors of ERS. ICA may protect neurons and promote the recovery of SCI through this way, but the specific mechanism is still unclear, which should be further verified by experiments. PC12 is a cell line derived from a pheochromocytoma of the rat adrenal medulla, not a stem cell, that have an embryonic origin from the neural crest that has a mixture of neuroblastic cells and eosinophilic cells. So we used PC12 cells as a neuron model for experiments.

## Methods

### Reagents and drugs

F-12 K medium (BOSTER, USA), fetal bovine serum (Life Technology 10,099,141, USA), horse serum, DMSO (Solebao, China), penicillin/streptomycin (Yuanpei, China), trypan blue dye Liquid (Melen, China), trypsin-EDTA, BCA protein content determination kit (Jin Yibai, China), poly-L-lysine (source leaf, China), nerve growth factor (Boaosen, China), NSE, MAP 2 antibody (abcam, USA), Grp78, CHOP antibody (Santa Cruz, USA), secondary antibody (Bio-Rad, USA), thapsigargin (TG), icariin (crystal pure, China), CCK8 kit (Biosharp, China), Sulfa Rhodamine B (SRB) Kit (Beibo, China), Annexin V-FITC Apoptosis Kit (Biyuntian, China), TaKaRa PrimeScriptTM RT Master Mix Reverse Transcription Kit, TaKaRa TB GreenTM Premix Ex Taq TMII PCR kit (TaKaRa, Japan) .

### Experimental grouping

The experiment was divided into 4 groups, blank group: PC12-induced differentiated neurons; DMSO group: PC12-induced differentiated neurons+ 0.1% DMSO; TG group: PC12-induced differentiated neurons+ 2 μmol/L TG (TG can induce ERS [18]); ICA group: neuronal cells of PC12 induced differentiation+ 2 μmol/L TG + 0.1 μmol/L ICA (preliminary experiments and literature review suggested that 0.1 μmol/L ICA can enhance the viability of PC12 cells [19]).

### Cell culture

The PC12 cells (which can be induced to differentiate into neurons by nerve growth factor) were purchased from Jiangsu Kaiji Biotechnology Co., Ltd., melted in a water bath at 37 °C. After centrifuging, F-12 K medium containing 10% horse serum+ 5% FBS + 1% penicillin/ streptomycin was added to resuscitate PC12 cells. After counting, 1 × 10^4^/ml cells were inoculated into 25cm^2^ petri dish and cultured at 37 °C in 5% CO_2_ incubator.

### Induced differentiation and identification of cells

PC12 cells with good growth were selected and inoculated into six-well plates coated with PLL, and nerve growth factor (NGF) was used to induce differentiation. After the protuberance of PC12 cells grew, 0.25% trypsin-EDTA was used to digest for 2 min. The PBS was used to resuscitate cells which were measured. There were 2–10 × 10^5^ cells in a EP tube, and they were divided into 2 groups with 3 tubes in each group. One group was added with 200uL NSE antibody, the other group was added with 200uL MAP 2 antibody. The mixture was gently blown and mixed, and incubated at 4 °C for 1 h. After centrifugation, 500uL cold PBS was used to wash it twice. Then the cells were transferred to the flow tube and detected by flow cytometry directly.

### CCK8 assay

PC12-induced differentiated neurons were counted after digestion, centrifugation and resuspension. According to the concentration of 1 × 10^4^/ml, 200 μL per well was inoculated into 3 96-well culture plates, and the acellular blank control group was set up parallel to the experiment. The culture plate was placed in the incubator for 24 h. After the cells adhered to the wall, each group was added to the drug-containing medium and cultured for 24 h, 48 h and 72 h, respectively. Instead of the original culture medium, F-12 K medium containing 10% horse serum+ 5% FBS + 1% penicillin/ streptomycin was added, while 10 μL CCK-8 solution was added to each well (avoid bubbles). The culture plates were placed in a static incubator for 4 h. Then the absorbance at 450 nm was determined by multi-function enzyme labeling instrument, and the difference was calculated.

### SRB assay

Neuron culture was the same as above. After 24 h, 48 h and 72 h, the 96-well plate was taken out, the culture medium was absorbed and discarded, and the follow-up steps were carried out according to the instructions of SRB assay kit. After fixation, washing, dyeing and incubation, the absorbance at 515 nm was determined by enzyme labeling instrument.

### Flow cytometry assay

The cells were intervened for 48 h. After digestion and centrifugation, the cells were suspended with binding buffer, mixed with MAP 2, and incubated without light for 1 h. The AnnexinV-FITC was added to incubate 10 min at room temperature without light. The cells were resuspended after washed for 3 times. The PI (final concentration was 1 μg/mL) was mixed and then flow cytometry assay was carried out.

### Transmission electron microscopic observation

The cells were intervened for 48 h. After digestion and centrifugation, the cells were fixed with 2.5% glutaraldehyde. Then the cells were fixed with 1% Osmic acid. After dehydration with different gradient concentrations of ethanol, it was soaked in different proportions of acetone and entrapment solution for several hours. After 3% uranium acetate-lead citrate double staining, the microstructure of the cells was observed under transmission electron microscope, and the ER structure of the cells was observed and evaluated.

### Western blot assay

The cells were intervened for 48 h. After cleavage with RIPA (including 1%PMSF), the total protein was obtained by centrifugation. After preparing separation gel and concentrated gel, electrophoresis was kept for 90 min with 100 V constant voltage. The PVDF film of appropriate size was cut, and transferred for 60 min with 100 V, 400 mA. Then the film was sealed at room temperature for 2 h by 5% skim milk. After TBST rinsing, CHOP, Grp78 and GAPDH antibodies were prepared according to the proportion of 1:1000, and incubated overnight at 4 °C. After rinsing, the second antibody was prepared according to 1:10000 and incubated at room temperature for 2 h. ECL developer was added and gel imaging system developed. The ImageJ image analysis system is used to analyze the strip and calculate the gray value of the strip.

### qPCR assay

The cells were intervened for 48 h. After digestion and centrifugation, RNA was extracted by Trizol method and the concentration of RNA in each group was determined. TaKaRa reverse transcription kit was used for reverse transcription. The sequences of CHOP, Grp78 and GAPDH were found on Genbank, and primers were designed and synthesized in Shanghai Shenggong. Using rat GAPDH as internal reference, the relative quantitative analysis of CHOP and Grp78 was carried out by TaKaRa TB GreenTM Premix Ex TaqTMII PCR kit. Applied Biosystems 7500 Fast Real-Time PCR System, was used to set the conditions of fluorescence quantitative PCR amplification for PCR reaction, and the value of 2^-ΔΔCt^ was calculated for relative quantitative analysis of the data.

### Statistical analysis

SPSS 20.0 software was used for statistical analysis. The data was shown as mean ± SD. One-way ANOVA and SNK-q test were used to analyze the differences among groups. The figures were edited by GraphPad Prism 8.0.2 software. A value of *P* < 0.05 was considered statistically significant.

## Results

### PC12 cells induced by NGF had neuron-like effect

**Fig. 1:**
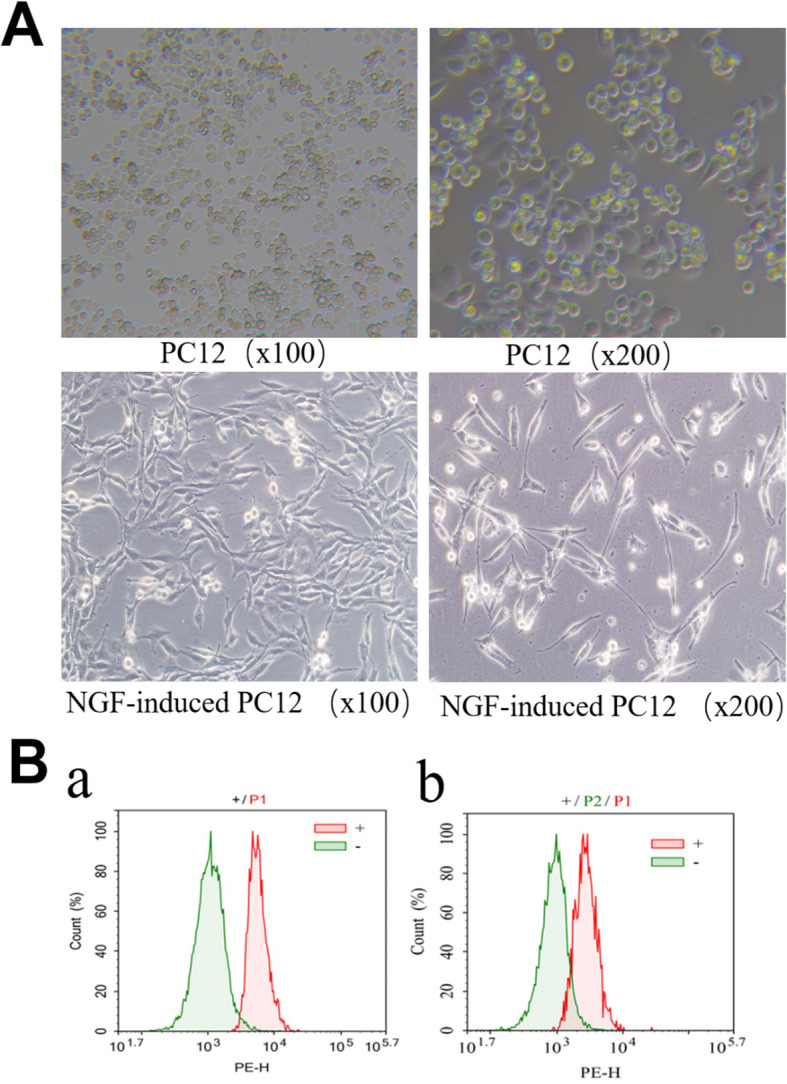
Morphology and flow cytometry identification of PC12 cells induced by NGF. **a** Inverted microscope was used to observe the PC12 cells and PC12 cells induced by NGF. After induction, the processes of PC12 cells became longer, and the cells grew in triangular and adherent shape. **b** The positive rates of MAP 2 and NSE were determined by flow cytometry. The results showed that the average positive rate of MAP 2 was 62.54% ± 1.00% (**a**) and the average positive rate of NSE was 93.32% ± 2.87% (**b**)

The PC12 cells induced by NGF for 7 days were observed by inverted microscope. The results showed that the induced PC12 cells had neuron-like morphology (Fig. 1a). Flow cytometry was used to detect the positive rate of MAP 2 and NSE expression. The results showed that PC12 cells induced by NGF had neuron-like effect and could be used in subsequent experiments (Fig. 1b).

### ICA promoted proliferation and reduced apoptosis of PC12 cells

**Fig. 2:**
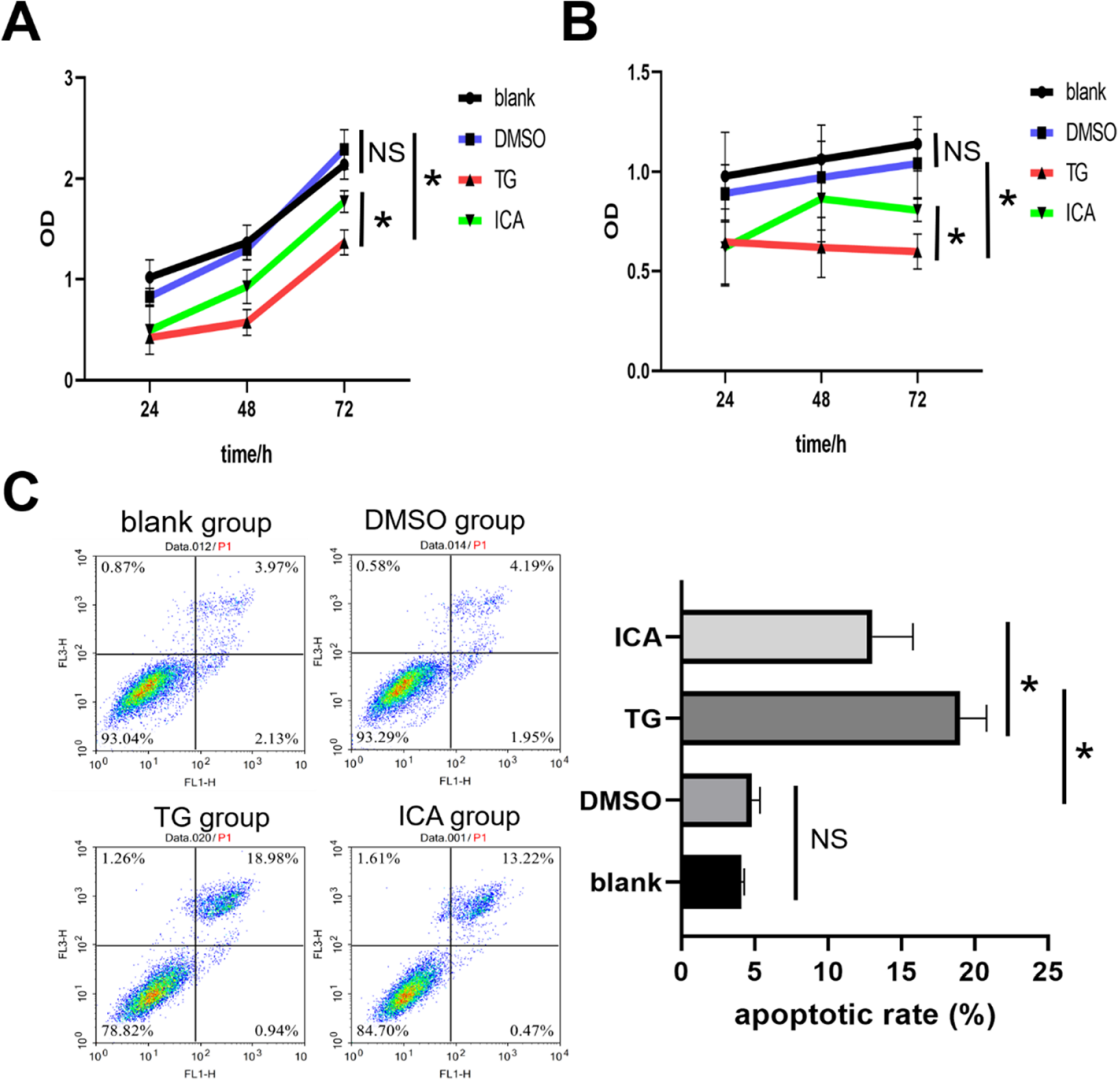
The results of CCK8 assay, SRB assay and flow cytometry assay. A The induced PC12 cells were cultured for 24 h, 48 h and 72 h, and CCK8 assay was carried out. The results showed that the OD value of each group increased gradually. At 72 h, there was no significant difference in OD value between DMSO group and blank group, OD value in TG group was significantly lower than that in DMSO group, and OD value in ICA group was significantly higher than that in TG group. B The induced PC12 cells were cultured for 24 h, 48 h and 72 h, and SRB assay was carried out. The results showed that the OD value of blank group and DMSO group increased gradually, but that of TG group showed a downward trend, and that of ICA group tended to be stable at 48 h. At 72 h, there was no significant difference in OD value between DMSO group and blank group, OD value in TG group was significantly lower than that in DMSO group, and OD value in ICA group was significantly higher than that in TG group. C The induced neurons were cultured for 48 h and double stained by AnnexinV-FITC and PI. The results showed that there was no significant difference in the apoptosis rate between the DMSO group and the blank group, the apoptosis rate in the TG group was significantly higher than that in the DMSO group, and the apoptosis rate in the ICA group was significantly lower than that in the TG group

After the induced PC12 cells were cultured for 24 h, 48 h and 72 h, CCK8 assay, SRB assay and flow cytometry assay showed that DMSO had no significant effect on PC12 cells, TG could inhibit proliferation and accelerate apoptosis of PC12 cells, but ICA could promote proliferation and reduce apoptosis of PC12 cells (Fig. 2).

### ICA improved the structure of ER in PC12 cells

**Fig. 3:**
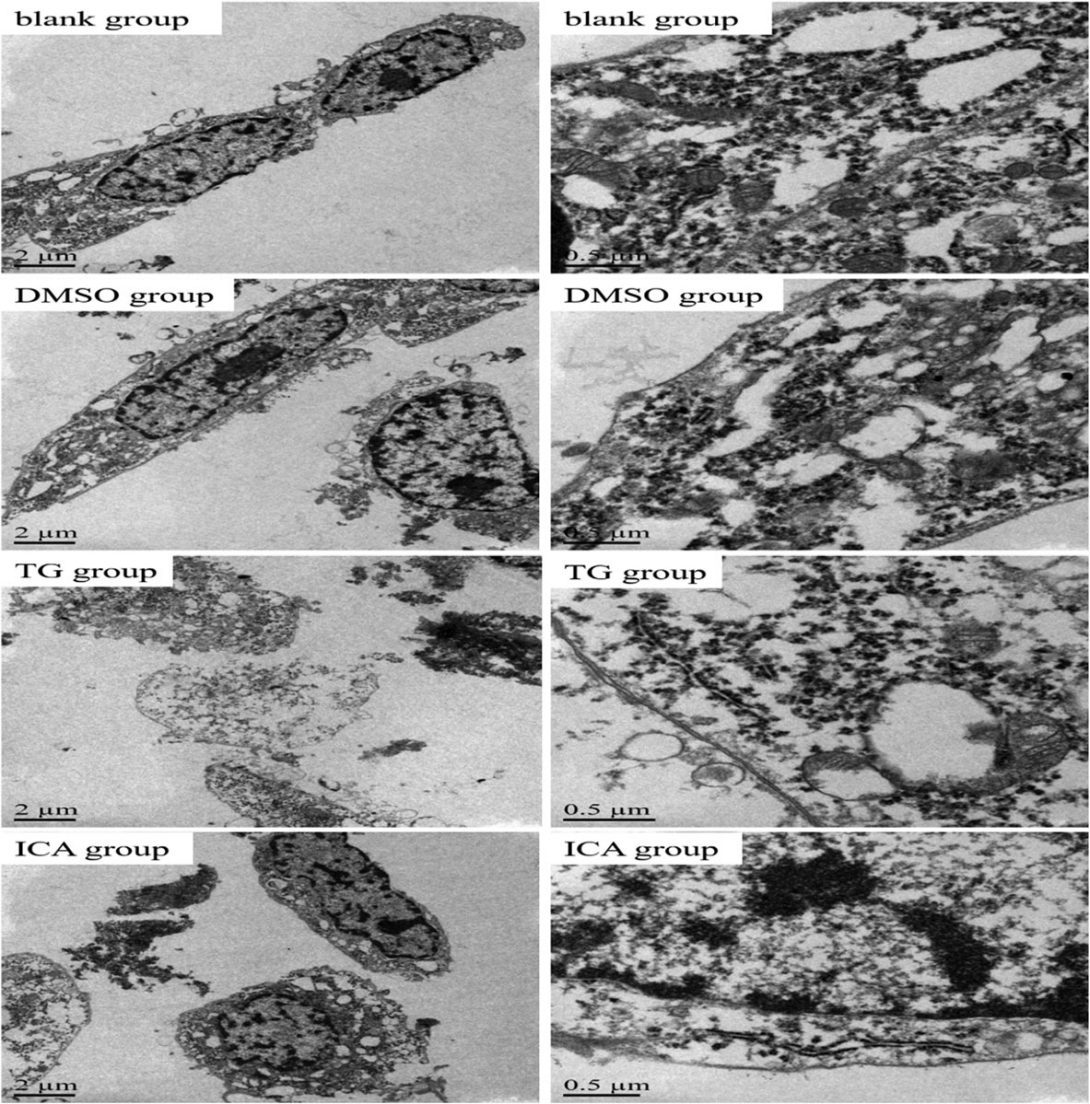
The microstructure of neuron ER. The PC12 cells of each group were observed by transmission electron microscope, and the results showed that there were abundant rough ER structures and nucleoli in blank group and DMSO group, vesicles of rough ER structure and partial nuclear fragmentation were observed in TG group, and the rough ER damage of neurons in ICA group was slighter

The neurons of each group were observed by transmission electron microscope, and the results showed that DMSO had no significant effect on the ER of PC12 cells, TG could destroy neuronal ER structure, but ICA could improve neuronal ER structure (Fig. 3).

### ICA reduced the expression of CHOP and Grp78 in PC12 cells

**Fig. 4:**
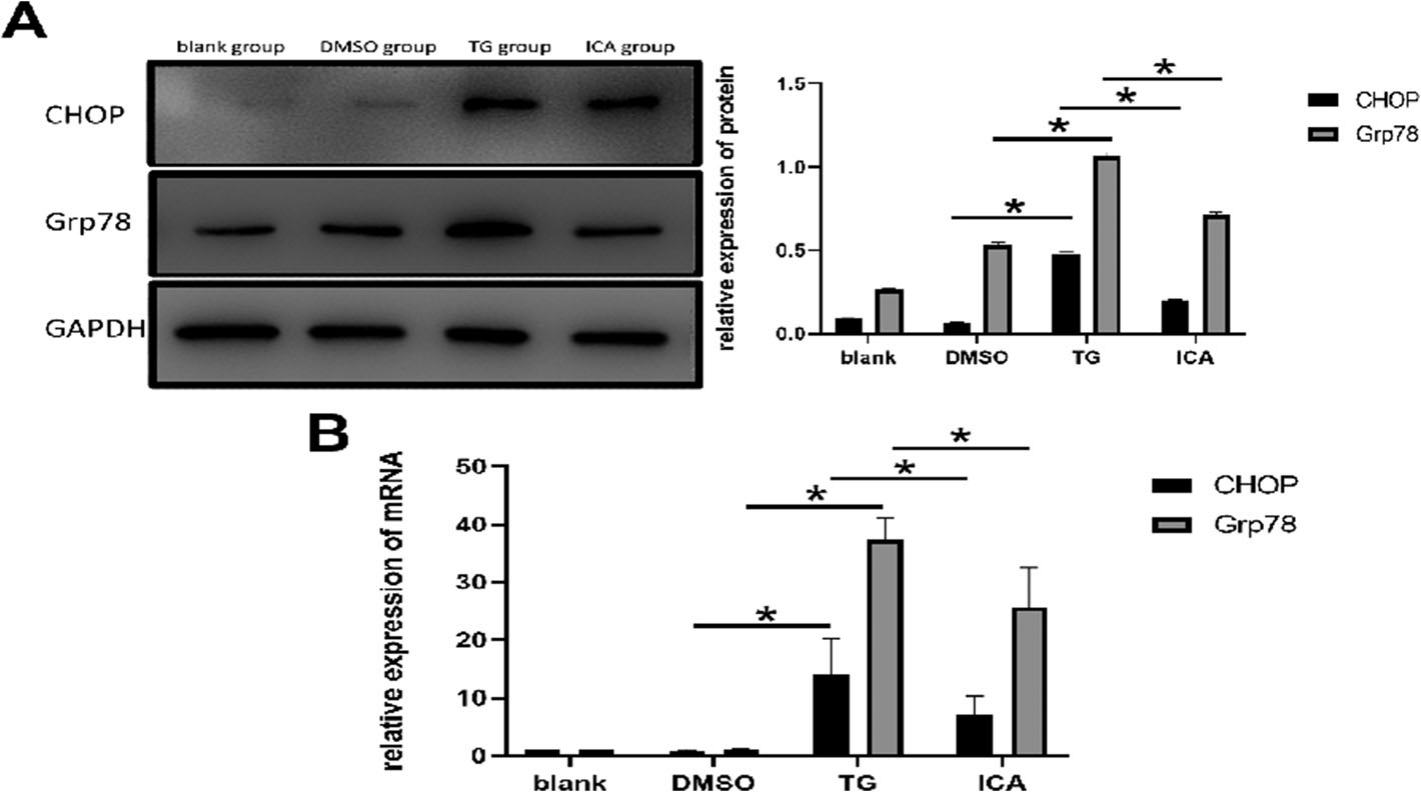
The results of Western blot and q-PCR assay. **a** The protein expression of CHOP and Grp78 in TG group was significantly higher than that in DMSO group. The protein expression of CHOP and Grp78 in ICA group was significantly lower than that in TG group. **b** The mRNA expression of CHOP and Grp78 in TG group was significantly higher than that in DMSO group. The mRNA expression of CHOP and Grp78 in ICA group was significantly lower than that in TG group

The results of Western blot and qPCR showed that TG could increase the expression of CHOP and Grp78, while ICA could decrease the expression of CHOP and Grp78, indicating that ICA could promote the recovery of PC12 cells by reducing the expression of CHOP and Grp78 (Fig. 4).

## Discussion

With the development of high-risk occupations and the increase in major accidents, the probability of SCI due to high energy is gradually increasing. SCI is a serious central nervous system disease, with many irreversible complications, and greatly reduces the quality of life of patients, but so far there is no better treatment, so it is worth exploring drugs for the treatment of SCI [20]. In recent years, Chinese medicine has developed rapidly and has been widely recognized internationally in some fields. Epimedium belongs to the Berberis family and is harvested when the stems and leaves are lush in summer and autumn, then removed thick stems and impurities, and dried or dried in the shade. In Chinese medicine, Epimedium has the effects of nourishing kidney yang, strengthening muscles and bones, and dispelling rheumatism. ICA is an extract of Chinese herbal medicine Epimedium, which can be used to treat SCI [21]. ICA may repair nerve tissue by regulating endoplasmic reticulum stress, but the specific mechanism is not clear.

Changes in the microenvironment of the SCI site can lead to protein misfolding [22, 23], and downregulation of ERS may reduce neuronal apoptosis and promote neurological recovery [8, 24, 25]. Some studies have shown that ICA can significantly reduce malondialdehyde content, increase superoxide dismutase activity, improve spinal lipid peroxidation, spinal cord edema and histopathological damage, and promote the recovery of motor function in rats with SCI [26]; Early and continuous treatment of high-dose ICA can inhibit pro-inflammatory factors, oxidative stress and neuronal apoptosis through the mitochondrial apoptotic pathway, and significantly promote exercise recovery after SCI [27]. Therefore, the effect of ICA on the repair of damaged neurons may be related to ERS.

In this study, ICA was used to interfere with PC12 cells to verify the effectiveness and partial mechanism of ICA regulating ERS to repair damaged neurons. The results of CCK8, SRB and flow cytometry assay showed that ICA could alleviate ERS induced by TG and decrease PC12 cells apoptosis. The observation of the microstructure of ER by transmission electron microscope showed that ICA could improve the apoptosis of PC12 cells induced by ERS. The results of Western blot and qPCR showed that ICA could inhibit ERS induced by TG through down-regulating the expression of CHOP and Grp78. According to the above data, ICA can inhibit ERS by down-regulating the expression of CHOP and Grp78, and promote the repair of PC12 cells.

Some studies have shown that up-regulation of Grp78 is beneficial to the correct folding of proteins in ER and promotes cell recovery, while down-regulation of Grp78 can cause accumulation of unfolded proteins in ER, and continuous activation of ERS leads to apoptosis [28, 29]. The high expression of CHOP indicates the activation of ERS and the trend of apoptosis in cells [30]. Other studies have shown that CHOP has anti-apoptotic effects [31]. In this experiment, ICA inhibited ERS and down-regulated the expression of CHOP and Grp78 in damaged neurons, thereby preventing neuronal apoptosis. This is different from the results of some literatures, there may be other pathways that affect the expression of CHOP and Grp78, so multi-experimental verification and multi-system pathway research should be carried out. However, this experiment still has some shortcomings, such as no animal experiments, no gene knockout, no systematic pathway research, the next step should be studied.

## Conclusions

ICA can inhibit ERS by down-regulating the expression of CHOP and Grp78, and promote the repair of PC12 cells. This study reveals part of the mechanism of ICA in the treatment of SCI and proves that ICA has the potential to promote the recovery of SCI.

## Supplementary Information

**Additional file 1.**

## Data Availability

The datasets used and/or analysed during the current study are available from the corresponding author on reasonable request.

## Abbreviations

ERS: Endoplasmic reticulum stress
ICA: Icariin
NGF: Nerve growth factor
SRB: Sulforhodamine B
CHOP: C/EBP homologous transcription factor protein
GRP78: Glucose regulation protein 78
IRE-1: Inositol-requiring enzyme 1
ATF6: Activating transcription factors 6
PERK: Protein kinase-like ER kinase
TG: Thapsigargin
