# Supplementary figures and images for: Icariin promotes the repair of PC12 cells by inhibiting endoplasmic reticulum stress
Source: BMC Complement Med Ther. 2021 Feb 19;21:69. doi: 10.1186/s12906-021-03233-1 (PMC7896365; doi:10.1186/s12906-021-03233-1)

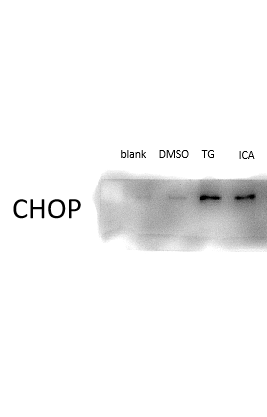

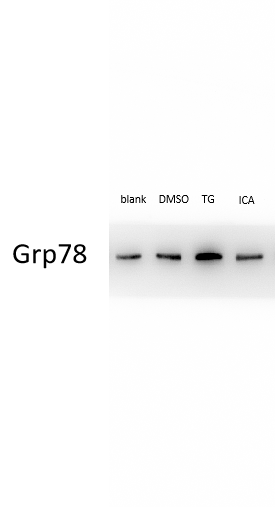

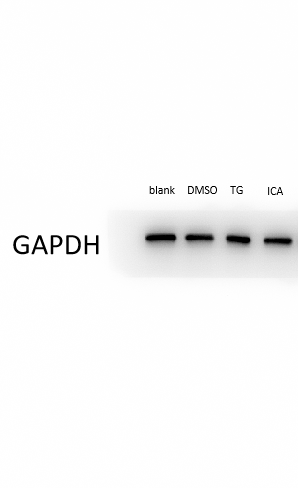

Supplement: Supplementary file 1 — Additional file 1. [file 12906_2021_3233_MOESM1_ESM.docx]
